# Phosphorus-Induced Lipid Class Alteration Revealed by Lipidomic and Transcriptomic Profiling in Oleaginous Microalga *Nannochloropsis* sp. PJ12

**DOI:** 10.3390/md17090519

**Published:** 2019-09-03

**Authors:** Jibei Liang, Sunya Iqbal, Fang Wen, Mengmeng Tong, Jianhua Liu

**Affiliations:** 1 Ocean College, Zhejiang University, Zhoushan 316000, China (J.L.) (S.I.) (F.W.); 2 School of Marine Science and Technology, Zhejiang Ocean University, Zhoushan 316022, China

**Keywords:** lipidomics, lipid class, *Nannochloropsis*, phosphate depletion, transcriptomics

## Abstract

Phytoplankton are primary producers in the marine ecosystem, where phosphorus is often a limiting factor of their growth. Hence, they have evolved strategies to recycle phosphorus by replacing membrane phospholipids with phosphorus-free lipids. However, mechanisms for replacement of lipid classes remain poorly understood. To improve our understanding, we performed the lipidomic and transcriptomic profiling analyses of an oleaginous marine microalga *Nannochloropsis* sp. PJ12 in response to phosphorus depletion (PD) and replenishing. In this study, by using (liquid chromatography couple with tandem mass spectrometry) LC-MS/MS-based lipidomic analysis, we show that membrane phospholipid levels are significantly reduced upon PD, while phosphorus-free betaine lipid levels are increased. However, levels of phosphorus-free photosynthetic galactolipid and sulfolipid are not increased upon PD, consistent with the reduced photosynthetic activity. RNA-seq-based transcriptomic analysis indicates that enzymes involved in phospholipid recycling and phosphorus-free lipid synthesis are upregulated, supporting the lipidomic analysis. Furthermore, enzymes involved in FASII (type II fatty acid synthesis) elongation cycle upon PD are transcriptionally downregulated. EPA (eicosapentaenoic acid) level decrease upon PD is revealed by both GC-MS (gas chromatography coupled with mass spectrometry) and LC-MS/MS-based lipidomic analyses. PD-induced alteration is reversed after phosphorus replenishing. Taken together, our results suggest that the alteration of lipid classes upon environmental change of phosphorus is a result of remodeling rather than de novo synthesis in *Nannochloropsis* sp. PJ12.

## 1. Introduction

Phytoplankton are primary producers that are believed to be responsible for nearly half of the global carbon-based photosynthesis [1,2]. Because of their relatively short generation time, they are very sensitive to the change of environmental stress conditions. In order to survive and propagate, they must have developed strategies to adapt from various environmental stress factors.

Phosphorus availability is a limiting factor of phytoplankton productivity in marine ecosystems [3]. Glycerophospholipid, a basic building block of plasma membranes, constitutes 10–20% of total cellular phosphorus, providing a potential pool for phosphorus recycling in case of phosphorus limitation. Numbers of studies including those using LC-MS/MS-based lipidomics have shown that phytoplankton have evolved a strategy of lipid remodeling to counter the effects of phosphorus deprivation by replacing phospholipids with phosphorus-free lipids [4,5,6].

As other stresses, phosphorus stress induces the decrease of cell growth rate in phytoplankton [7,8]. It is found to be accompanied with shrunk chloroplast and decreased photosynthetic efficiency [9]. Chloroplast contains unique thylakoid membrane-associated lipids phosphatidylglycerol (PG), digalactosyldiacylglycerol (DGDG), monogalactosyldiacylglycerol (MGDG), and sulfoquinovosyldiacylglycerol (SQDG) [10]. After chloroplast shrinking, excess plastid membrane lipids are the potential pool for recycling phosphorus from PG and conversion into neutral lipid TAG (triacylglycerol) [9].

Besides plastid glycerophospholipid PG, PC (phosphatidylcholine) and PE (phosphatidylethanolamine) are major non-plastid glycerophospholipid in phytoplankton. Notably, non-plastid phosphorus-free betaine lipid DGTS (diacylglyceryltrimethylhomo-ser) is analogous to PC and PE [11], making it ideal for replacing PC and PE under phosphorus limited conditions. It has been proposed that conversion between DGTS and PC or PE undergoes through do novo synthesis after breakdown (or “switching”) or modification of glycerolipid head (or “remodeling”) [12,13,14].

Transcriptomic profiling analysis has been widely used for the study of phenotypic changes during response to various environmental stress conditions [15,16,17]. Enzymes in algae and higher plants appear to be encoded by multiple-copy genes [18,19,20,21]. To predict whether the accumulated TAG is synthesized in ER or plastid, a number of algorithms attempt to predict subcellular localizations of the relevant enzyme isoforms [22,23,24,25,26]. However, the prediction is not always unambiguous. On the other hand, in algae and higher plants, glycerolipid synthesis pathways in ER (or the “eukaryotic” pathway) and plastid (or the “prokaryotic” pathway) can be distinguished by the fatty acids 18:X and 16:X at the *sn*2 position of the glycerol backbone, respectively [27,28,29,30,31]. Hence, by combining the transcriptomic and lipidomic analyses, alteration of lipid classes under phosphorus limitation can be better revealed.

To investigate alterations of lipid classes and regulation of respective enzymes in oleaginous alga *Nannochloropsis* sp. (Phylum *Ochrophyta*, Class *Eustigmatophyceae*) PJ12, we conducted the LC-MS/MS-based lipidomic analysis and RNA-seq transcriptomic profiling. Our results show that, upon phosphorus depletion, levels of unique thylakoid membrane-associated lipids DGDG, MGDG, PG, and SQDG and major non-plastid glycerophospholipids PC and PE are decreased, while level of non-plastid phosphorus-free betaine lipid DGTS, analogous to PC and PE, is increased. Transcriptional change of respective enzymes support the level change of the lipid classes.

## 2. Results

### 2.1. Phosphate Depletion and Restoration in Nannochloropsis sp. PJ12 Cultures

**Figure 1:**
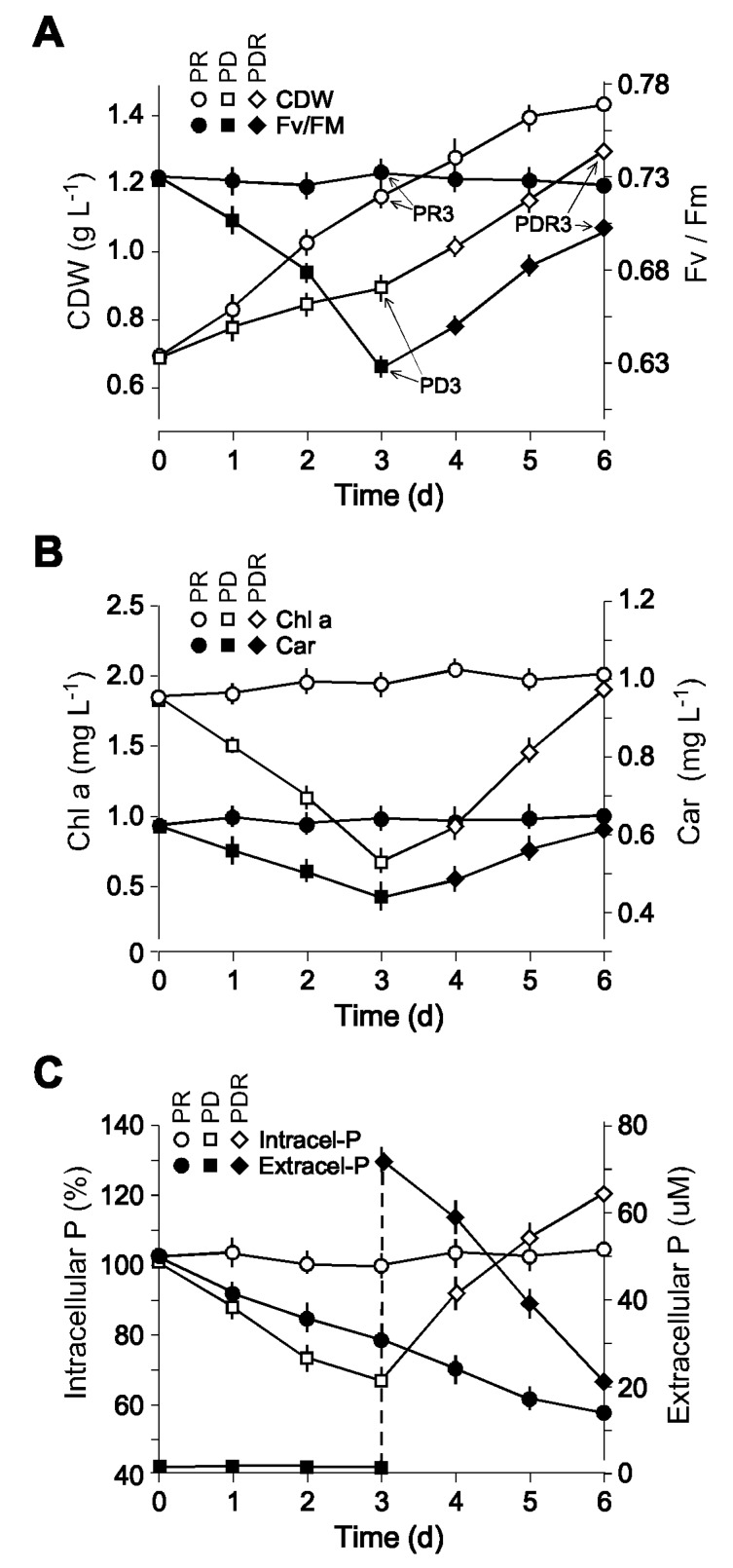
Characteristics of cell growth, photosynthetic efficiency, light harvesting pigments, and intra and extracellular phosphorus of PJ12 cultures upon phosphate depletion and restoration. (**A**) Cell growth and photosynthetic efficiency. Cell growth is determined gravimetrically. CDW stands for cell dry weight. Photosynthetic efficiency (Fv/Fm) is estimated based on PAM fluorescence. PR3, PD3, and PDR3 indicate the sampling points for cells under phosphate replete, phosphate depleted, and phosphate restored samples. (**B**) Level of light harvesting pigments chlorophyll a and carotene. Pigments are determined using colorimetric methods. (**C**) Level of intracellular and extracellular phosphorus. Phosphorus concentration is determined colorimetrically.

*Nannochloropsis* sp. PJ12 cell were grown in flask containing f/2 medium [32] with orbital shaking at 100 rpm under continuous light of 50 µM photons m^−1^ s^−1^ at room temperature (25 °C). Culture reaching log-phase at the OD ~ 0.7 was subjected to medium shift to phosphate-depleted medium (i.e., f/2-P) (Figure 1A). As a control, an equal portion of cells was grown in phosphate-replete medium (i.e., f/2). Cell growth and photosynthetic efficiency (Fv/Fm) were monitored in triplicate by gravimetric method and pulse amplitude modulated (PAM) fluorimetric technology, respectively. We found that cell growth in terms of cell mass increase was impeded when cultivated in f/2-P medium (Figure 1A, see open square). This was correlated with the decreased photosynthetic efficiency (Figure 1A, see open closed square). As a control, cell mass was found to increase by 1.67-fold in three days, during which the photosynthetic efficiency remained constant (Fv/Fm = 0.74). After phosphate restoration, we found that cell mass increased again. At day 3 after phosphate restoration, cell biomass was found to increase by 1.65-fold. This was correlated with the increased photosynthetic efficiency. Cells 3 days after growth in phosphate-replete medium f/2 and phosphate-depleted medium f2-P were designated as PR3 and PD3 (respectively), while 3 days after phosphate-restoration of PD3 was designed as PDR3 (Figure 1A, see PR3, PD3, and PDR3).

Subsequently, we determined chlorophyll *a* and carotenoid contents in cells under phosphate replete (PR3), depleted (PD3), and restored (PDR3) conditions. Levels of both chlorophyll *a* and carotenoid contents were correlated with the level of photosynthetic efficiencies (Figure 1B).

Intracellular and extracellular phosphorus contents were determined using colorimetric methodology in triplicate (see Materials and Methods). Intracellular phosphorus content in cells at various time points including PR3 during growth under phosphate-replete conditions was nearly constant (Figure 1C). Hence, the intracellular phosphorus concentration of PR3 was set as 100%. In contrast, intracellular phosphorus content of PD3 was decreased to 69% compared to that of PR3. We noted that the phosphorus contents in PDR3 was increased to 120% compared to that of PR3, suggesting that phosphorus restoration after depletion led the cells over-uptake phosphorus. 

### 2.2. Alteration of Fatty Acid Profiles upon Phosphate Depletion and Restoration in PJ12 Cells

**Figure 2:**
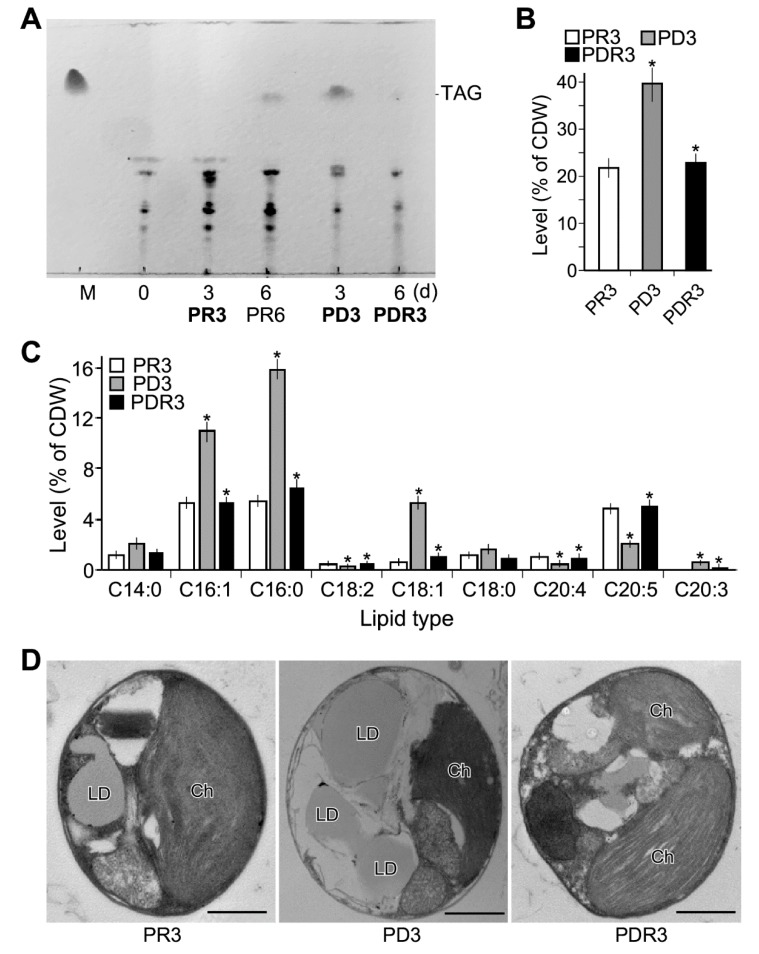
Characteristics of lipid composition in PJ12 cells prior to and after phosphate depletion and restoration. (**A**) TLC analysis of total lipid extracted from PJ12 cells. Samples in bold PR3, PD3, and PDR3 are subjected to transcriptomic and lipidomic analysis in triplicate. (**B**) Level of total acyl lipids. Asterisk sign (*) indicates the significance of level difference between PD3 and PR3, and between PDR3 and PD3. (**C**) GC analysis of total acyl lipids as FAME (fatty acid methyl esters). Asterisk sign (*) indicates the significance of level difference between PD3 and PR3, and between PDR3 and PD3. (**D**) Transmission electron microscope. LD and Ch stand for lipid droplet and chloroplast, respectively. A scale bar of 1 μm is indicated.

To investigate the lipid content changes in cells after phosphate deprivation and restoration, we determined the lipid contents of PR3, PD3, and PDR3 cells. Total lipids were extracted in triplicate using chloroform: methanol (2:1 *v*/*v*) solution based on the method previously reported [33]. Thin-layer chromatography (TLC) analysis indicated that triacylglycerol (TAG) was hardly detected in total lipid extracted from PR3 cells (Figure 2A). In contrast, TAG was found to be 40% of total lipids derived from PD3 cells. Upon phosphorus restoration, TAG content was found to be 10% of total lipid from PDR3 cells. Total acyl lipids as fatty acid methyl esters (FAME) were 21%, 39%, and 23% of CDW in PR3, PD3, and PDR3 cells, respectively (Figure 2B).

GC-MS analysis indicated that nine species of fatty acids were detected in PR3, PD3, or PDR3 cells (Figure 2C). We found that palmitic acid (C16:0), palmitoleic acid (C16:1n-7), and eicosapentaenoic acid or EPA (C20:5n-3) were the top three most abundant fatty acids in PR3 and PDR3 cells. On the other hand, palmitic acid (C16:0), palmitoleic acid (C16:1n-7), and oleic acid (C18:1n-9) were the top three most abundant fatty acids in PD3 cells, all of which were significantly increased compared to that of PR3 and PDR3 cells (level change > 2-fold, *p-*value < 0.05, *n* = 3). But the level of EPA (C20:5n-3) in PD3 cells was significantly decreased compared to that of PR3 and PDR3 cells (level change > 2-fold, *p*-value < 0.05, *n* = 3). These results suggested that palmitic acid (C16:0), palmitoleic acid (C16:1n-7), oleic acid (C18:1n-9), but not EPA (C20:5n-3) were the major constituents of TAG in PD3 cells.

EM analysis indicated that numbers of oil droplets were higher in PD3 cells compared to that of PR3 and PDR3 cells (Figure 2D). Meanwhile, shrinking of chloroplast was apparent in PD3 cells compared to others, consistent with the decreased photosynthetic efficiency observed (see Figure 1A).

### 2.3. Lipidomic Profiling of PJ12 upon Phosphate Depletion and Restoration

**Figure 3:**
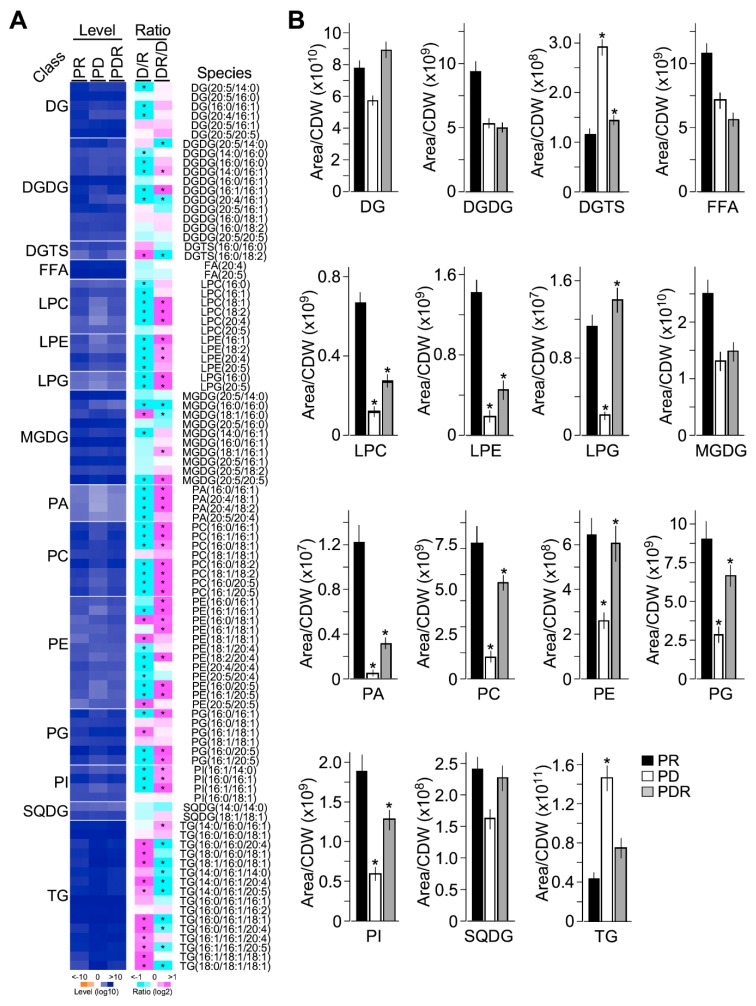
Lipidomic profiles of PR3, PD3, and PDR3 samples. (**A**) Level and ratio of 95 lipid molecules found in JP12 in triplicate. Average level and ratio of each molecule as indicated are shown. Asterisk (*) indicate the significant change of levels (level change > 2-fold, *p*-value < 0.05, *n* = 3). (**B**) Level of 15 lipid classes as indicated. Asterisk (*) indicate the significant change of levels (level change > 2-fold, *p*-value < 0.05, *n* = 3).

Based on lipidomic analysis using LC-MS/MS technology, we identified a total of 95 lipid species in 15 lipid classes including DGDG (digalactosyldiacylglycerol), DGTS (diacylglyceryltrimethylhomoserine), FFA (free fatty acid), LPC (lysophosphatidylcholine), LPE (lysophosphatidylethanolamine), LPG (lysophosphatidylglycerol), MGDG (monogalactosyldiacylglycerol), PA (phosphatidic acid), PC (phosphatidylcholine), PE (phosphatidylethanolamine), PG (phosphatidylglycerol), PI (phosphatidylinositol), and SQDG (sulfoquinovosyl diacylglycerol) in negative mode and DG (diacylglycerol) and TG (triacylglycerol) in positive mode, in which lipid levels were estimated based on pixels of peak area per cell (or million cells) (Figure 3A, Table S1) (see Materials and Methods). Of 95 lipid species, 68 (73.1%) were found to exhibit significant level change (level change > 2-fold, *p*-value < 0.05) upon phosphate depletion and/or phosphate restoration.

All classes of phospholipids LPC, LPE, LPG, PA, PC, PE, PG, and PI whose levels exhibited significant decrease and increase (level change > 2-fold, *p*-value < 0.05, *n* = 3) upon phosphate depletion and restoration, respectively (Figure 3B). Levels of phosphorus-free lipids DGDG, MGDG, and SQDG showed no significant alteration at the level of 2-fold change, but at the level of 1.5-fold change (*p*-value < 0.05, *n* = 3). Notably, phosphorus-free lipid DGTS was significantly increased and decreased (level change > 2-fold, *p*-value < 0.05, *n* = 3) upon phosphate depletion and restoration, respectively. This result was consistent with the notion that DGTS was analogous with phospholipids PC and PE and replaced PC and PE under phosphate limited conditions [11,34].

On the other hand, levels of neutral lipid TG were greatly increased upon phosphate depletion and decreased upon restoration (level change > 2-fold, *p*-value < 0.05, *n* = 3). Levels of phosphorus-free lipids such as DGDG, MGDG, and SQDG were not increased to replace the loss of phospholipid. This result would suggest that plastid membrane lipids DGDG, MGDG, and SQDG were partly converted to non-plastid membrane lipid DGTS upon PD.

### 2.4. Common DE Genes Are Phosphorus-Specific Response Genes

**Figure 4:**
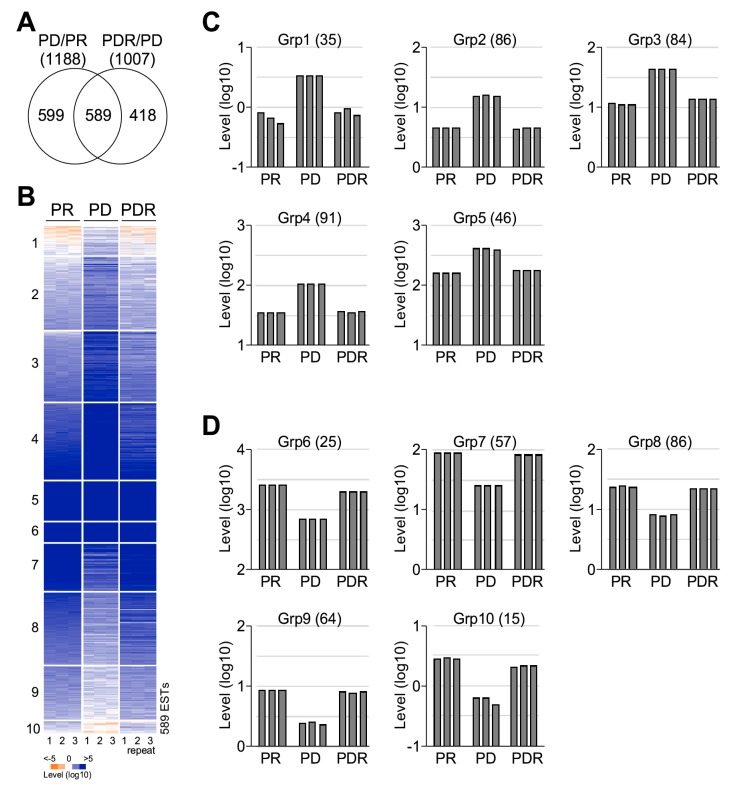
Common DE (differentially expressed) genes are phosphate-specific response genes. (**A**) Venn diagram showing the common DE genes between the DE gene upon phosphate depletion (PD/PR) and the DE genes upon phosphate restoration (PDR/PD). (**B**) K-mean 10-group clustering analysis of the common DE genes. (**C**) K-mean groups containing DE genes whose transcription level change inversely correlated with that of phosphate concentrations. (**D**) K-mean groups containing DE genes whose transcription level change positively correlated with that of phosphate concentrations.

We performed RNA-seq-based transcriptomic analysis with a total of 18 Gigabase short read sequences (see Materials and Methods). As a result, a transcriptome containing 7140 annotated ESTs or genes (they were simply referred as genes hereafter) without a single missing value from nine measurements under three different growth conditions: PR3 (or PR), PD3 (or PD), and PDR3 (or PDR) (see Figure 1A, Table S2). Transcription levels were normalized to TPM (transcripts per million) for comparison between different conditions. Of the total of 7140 genes, we found 1188 (16.6%) differentially expressed (DE) genes (level change > 2-fold, *p*-value < 0.05) between PR and PD conditions. On the other hand, there were 1007 (14.1%) DE genes (level change > 2-fold, *p*-value < 0.05) between PD and PDR conditions. Notably, 589 genes were found in both sets of DE genes (*p*-value < 2.2 × 10^−16^), indicating that these common DE genes were responsive to both decrease and increase of phosphate in growth medium (Figure 4A).

The common set of 589 genes was subsequently subjected to the K-mean 10-group clustering analysis (Figure 4B, Table S3) using Cluster [35]. We found that transcriptional levels of the first five Groups (Group 1 to Group 5) displayed an inverse correlation with that of the phosphate concentrations in medium: Transcriptional levels in cells under PR and PDR conditions were lower than that under PD condition (Figure 4C). In contrast, transcriptional level of the second five Groups (Group 6 to Group 10) showed a positive correlation with that of the phosphate concentrations (Figure 4D). These results would suggest that the common DE genes were phosphorus-specific response genes in *Nannochloropsis* sp. PJ12.

Functional enrichment analysis of KEGG (Kyoto Encyclopedia of Genes and Genomes) metabolic pathways indicated that genes (in Group 1 to Group 5) whose transcription levels were inversely correlated with concentrations of phosphate were enriched for a KEGG metabolic pathway, cysteine, and methionine metabolism (level of enrichment > 2-fold, *p*-value < 0.05, number of the pathway-associated genes >= 5) (see Materials and Methods). On the other hand, genes (in Group 6 to Group 10) whose expression levels were positively correlated with the phosphate concentrations were enriched for 84 KEGG metabolic pathways (level of enrichment > 2-fold, *p*-value < 0.05, number of associated genes >= 5), the top 10 of which were listed in Table 1 (for a full list, see Table S4).

### 2.5. Coherently Transcriptional Change of Enzymes Involved in Calvin Cycle but not Glycolysis or TCA (tricarboxylic acid) Cycle upon Phosphate Depletion and Restoration

**Figure 5:**
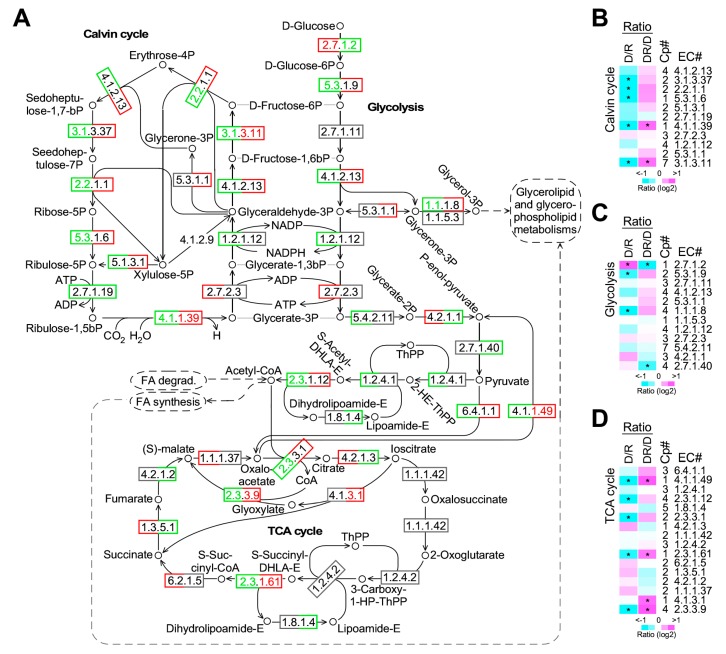
Transcriptional profiling of enzymes involved in Calvin cycle, glycolysis, and TCA cycle. (**A**) A schematic drawing of the Calvin cycle, glycolysis, and TCA cycle. EC number of enzymes and metabolites are indicated. Differentially increased and decreased (level change > 1.5-fold, *p*-value < 0.05) enzymes whose EC number are shown in red and green, respectively. Moderately increased and decreased (level change > 10%) enzymes whose rectangle number box are shown in red and green, respectively. Enzymes not present in the transcriptome are shown without box. Left and right portions of EC numbers indicate the response to phosphate depletion (PD/PR) and phosphate restoration (PDR/PD), respectively. (**B**) Transcriptional response of enzymes involved in Calvin cycle. Level increase and decrease are indicated by magenta and cyan, respectively. Copy number (Cp#) of enzyme-coding genes are indicated. Asterisk (*) indicate the significant level change (level change > 1.5-fold, *p*-value < 0.05). (**C**) Transcriptional response of enzymes involved in glycolysis. The display is identical to (**B**). (**D**) Transcriptional response of enzymes involved in TCA cycle. The display is identical to (**B**).

Of the 35 unique enzymes involved in Calvin cycle, glycolysis, and TCA cycle, the majority (77.1%) were encoded by two- or multi-copy genes (Figure 5A, Table S5). Hence, in this analysis, transcriptional level of enzymes was based on the sum level of multi-copy genes when present (see Materials and Methods). Based on this approach, we investigated the consistence of transcriptional level changes of enzymes in a pathway upon phosphate depletion and restoration. We defined an enzyme whose transcriptional level was greatly altered and moderately altered if the level change was greater than 1.5-fold (*p*-value < 0.05) and 10% upon phosphate depletion and restoration, respectively (see Materials and Methods). In Calvin cycle, we found that nine out of ten moderately altered (level change > 10%) enzymes were downregulated upon phosphate deprivation (*p* = 0.011), five of which were significantly downregulated (level change > 1.5-fold, *p*-value < 0.05) (Figure 5B, see D/R). In contrast, eight out of nine moderately altered (level change > 10%) enzymes involved in Calvin cycle upon phosphate restoration were upregulated (*p*-value = 0.019) (Figure 5B, see DR/D). These results suggested that the activity of enzymes involved in Calvin cycle was concordantly downregulated and upregulated under phosphate-depleted and -restored conditions (respectively), consistent with the change of cell growth rate and photosynthetic efficiency (see Figure 1A).

However, transcriptional change of enzymes involved in glycolysis was a complex: Five and three out of twelve enzymes involved in glycolysis were moderately downregulated and upregulated (level change > 10%) upon phosphate depletion, respectively (Figure 5A,C, see D/R), showing no consistent transcriptional change. Likewise, four and three out of the twelve enzymes were upregulated and downregulated (level change > 10%) after phosphate restoration, respectively (Figure 5A,C, see DR/D). A similar complex pattern was also observed for enzymes involved in TCA cycle (Figure 5D). 

### 2.6. TAG Accumulation in Both Plastid and Cytosol upon Phosphate Depletion

**Figure 6:**
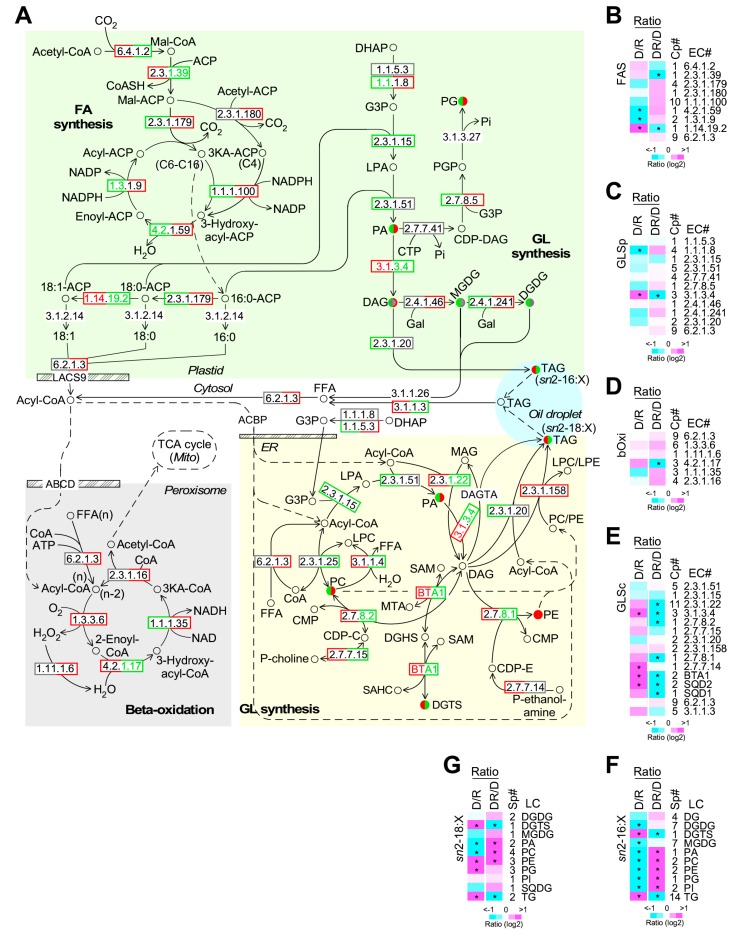
Transcriptional profiling of enzymes involved in type II fatty acid synthesis (FASII), glycerolipid synthesis in plastid (GLSp) and cytosol glycolysis (GLSc), and beta-oxidation. (**A**) A schematic drawing of the FASII, GLSp, GLSc, and beta-oxidation. Metabolites whose level increase or decrease is shown in red and green, respectively. Significant (level change > 1.5-fold, *p*-value < 0.05) and moderate (level change > 10%) changes are indicated by fill and border, respectively. Left and right portions present the change upon phosphate depletion (PD/PR) and phosphate restoration (PDR/PD), respectively. The display is identical to Figure 5A. (**B**) Transcriptional response of enzymes involved in FASII. The display is identical to Figure 5B. (**C**) Transcriptional response of enzymes involved in GLSp. The display is identical to Figure 5B. (**D**) Transcriptional response of enzymes involved in beta-oxidation. The display is identical to Figure 5B. (**E**) Transcriptional response of enzymes involved in GLSc. The display is identical to Figure 5B. (**F**) Level change of metabolites involved in the GLSp. It is synthesized through the “prokaryotic” pathway. (**G**) Level change of metabolites involved in the GLSc. It is synthesized through the “eukaryotic” pathway.

We subsequently investigated the transcriptional profile of enzymes involved in the type II fatty acid synthesis (FASII), glycerolipid synthesis in plastid (GLSp) and cytosol or ER (GLSc), and beta-oxidation in peroxisome (Figure 6A, Table S6). Based on the transcriptomic profiling, we found that all four enzymes involved in elongation cycle of FASII were downregulated and upregulated (level change > 10%) upon phosphate-depletion and phosphate restoration, respectively (Figure 6A and 6B). However, enzymes involved in glycerolipid synthesis in plastid and beta-oxidation in peroxisome showed no concordant alteration of transcriptional levels upon phosphate depletion and restoration (Figure 6C,D). On the other hand, in the glycerolipid synthesis in cytosol, eleven out of 14 moderately altered (level change > 10%) enzymes were upregulated upon phosphate depletion (*p*-value = 0.028) (Figure 6E, see D/R). Additionally, twelve out of 13 moderately altered (level change > 10%) enzymes were downregulated upon phosphate restoration (*p*-value = 0.0017) (Figure 6E, see DR/D). This was consistent with the observation that TAG level increased and decreased upon phosphate depletion and restoration, respectively (see Figure 3B).

Based on the lipidomic profiling analysis, levels of “prokaryotic pathway” synthesized lipids (i.e., synthesized in plastid with a C16:X at the *sn*2-position of the glycerol backbone) PA, PC, PE, and PG were significantly decreased and increased (level change > 1.5-fold, *p*-value < 0.05, *n* = 3) upon PD and PDR, respectively (see Figure 6F). In contrast, levels of DGTS and TG showed the opposite change, suggesting that plastid synthesized lipids PA, PC, PE, and PG were modified into DGTS and TG upon PD and reversed upon PDR. 

Level changes of the “eukaryotic pathway” synthesized lipids (i.e., synthesized in cytosol/ER with a C18:X at the *sn*2-position of the glycerol backbone) such as PA, PC, DGTS, and TG resembled that of “prokaryotic pathway” synthesized ones. However, levels of the “eukaryotic pathway” synthesized PE and PG were increased upon PD (level change > 1.5-fold, *p*-value < 0.05, *n* = 3) (see Figure 6G), displaying an opposite change to that of the “prokaryotic pathway” synthesized PE and PG.” 

### 2.7. Level of EPA in PC and PE is Decreased and Increased upon Phosphate Depletion and Restoration

**Figure 7:**
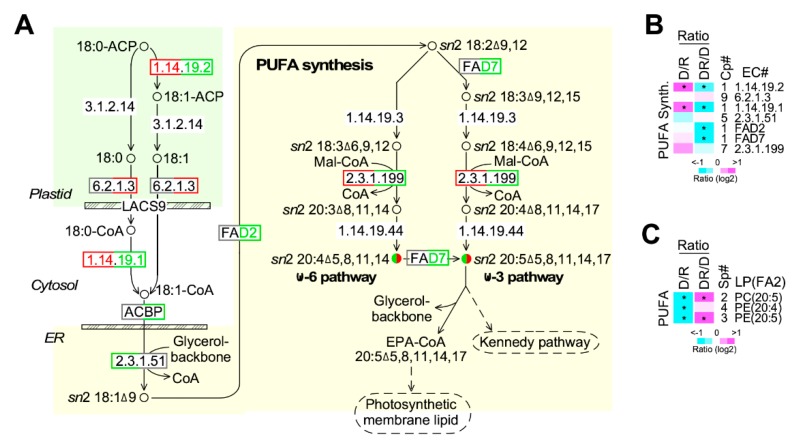
Transcriptional profiling of enzymes involved in PUFA synthesis. (**A**) A schematic drawing of the PUFA synthetic pathway. The display is identical to Figure 6A. (**B**) Transcriptional response of enzymes involved in PUFA synthetic pathway. The display is identical to Figure 5B. (**C**) Level change of metabolites involved in PUFA synthetic pathway. The display is identical to Figure 6F.

We investigated the transcriptional profile of enzymes involved in PUFA (polyunsaturated fatty acids) synthesis (Figure 7A, Table S7). Based on the sum level of multi-copy genes when existed, we found that transcription levels of enzymes involved in PUFA synthesis appeared to be increased and decreased upon phosphate depletion and restoration, respectively (Figure 7B). However, based on the lipidomic profile, level of PC and PE whose *sn*2 position of the glycerol backbone was esterified with EPA was greatly decreased and increased (level change > 1.5-fold, *p*-value < 0.05) upon phosphate depletion and restoration, respectively (Figure 7C). It was noted that level of PE containing esterified ARA (arachidonic acid) at the *sn*2 position of the glycerol backbone was significantly decreased (level change > 1.5-fold, *p*-value < 0.05) upon phosphate depletion and only moderately increased (level change > 10%) on phosphate restoration. These results were consistent with the GC-MS-based analysis (see Figure 2B).

## 3. Discussion

In this study, we show that growth rate and photosynthetic efficiency of *Nannochloropsis* sp. PJ12 are decreased upon the shift from phosphorus-replete (PR) condition to phosphorus-depleted (PD) condition (see Figure 1A). The growth rate and photosynthesis are restored after shifting back from PD condition to PR condition. This is consistent with the proposal that phytoplankton has adapted a strategy of lipid remodeling to counter the effects of phosphorus stress by replacing the membrane phospholipids with phosphorus-free lipids [13,36,37].

Responsive genes identified based on the transcriptional profiling analysis are not necessarily specific to the stress factor applied [38]. Responsive genes specific to tress factors should exhibit transcriptional alteration correlated with the dosage of stress factors [21,39]. Based on transcriptional profiling in response to reciprocal alteration of phosphate concentrations, we show, in this study, 589 genes display differentially transcriptional responses (level change > 2-fold, *p*-value < 0.05) upon the shift from PR condition to PD condition and shift from PD condition to PR (or PDR) condition. Approx. 42% and 58% of the 589 common DE genes are positively and negatively correlated with the change of phosphate, respectively (see Figure 4). This subset of ED genes is likely to be the phosphate-specific responsive genes in *Nannochloropsis*. sp. PJ12, which enriches many enzymes involved in various metabolic pathways (see Table 1).

Upon stress, concordantly transcriptional change of enzymes would direct a metabolic flux in a pathway. It has been shown that nitrogen deprivation leads to the consistent downregulation of transcription of nearly all enzymes (*p*-value < 0.05) involved in Calvin cycle, glycolysis, and TCA cycle in *Nannochloropsis* sp. PJ12 [33]. In this study, we show that, based on the transcriptional profiling, enzymes involved only in Calvin cycle, but not glycolysis or TCA cycle, are concordantly downregulated and upregulated upon phosphate depletion and restoration, respectively (see Figure 5). This result would suggest that phosphate limitation reduces photosynthetic sugar production, consistent with the reduction (i.e., ~ 15%) of photosynthetic efficiency (see Figure 1). The effect is clearly reversed after phosphate restoration.

It has been shown that upon nitrogen limitation, reduction of photosynthetic efficiency in PJ12 is accompanied with the decrease of major plastid phosphorus-free lipids DGDG and phospholipid PG (level change > 2-fold, *p*-value < 0.05, *n* = 3), but not the non-plastid phospholipid PC and PE [33]. In this study, we show that, upon phosphate deprivation, level of all membrane phospholipids including non-plastid lipids PA, PC, and PE and plastid lipid PG is reduced (level change > 2-fold, *p*-value < 0.05, *n* = 3) in PJ12, while the level of the non-plastid phosphorus-free betaine lipid DGTS is increased (see Figure 3). The level of plastid phosphorus-free galactolipids DGDG and MGDG is moderately decreased (level change > 1.5-fold, *p*-value < 0.05, *n* = 3) under phosphate limitation, unlike non-plastid phosphorus-free lipid DGTS. This is consistent with the reduced photosynthetic efficiency. These results suggest that upon phosphorus-depletion, not only the membrane phospholipids (PA, PC, PE, and PG) are converted into phosphorus-free lipids (DGTS), but also the plastid phosphorus-free lipids (DGDG, MGDG, and SQDG) are converted into non-plastid lipids (DGTS) in *Nannochloropsis* sp. PJ12. 

It is known that PC, PE, and PG are involved in the Lands’ cycle lipid modification pathway on ER [40]. In this study, we show that levels of “prokaryotic pathway” synthesized PE, and PG, similar to other phospholipids, are decreased and increased upon PD and DPR, respectively, correlated with the change of phosphorus concentrations. However, levels of “eukaryotic pathway” but not the “prokaryotic pathway” synthesized PE and PG are significantly increased upon PD, suggesting that PE and PG in PJ12 might be involved in Lands’ cycle lipid remodeling pathway [40].

It has been shown that *N. salina* is capable of over uptake phosphorus for storage under phosphate replete condition [41]. This may be partly attributed to its resistance to phosphorus deprived condition, besides the phospholipid replacement by the phosphorus-free lipids.

Transcriptomic profiling analysis shows that, in this study, many enzymes involved in phosphate recycling such as phosphatidic acid phosphatase (EC 3.1.3.4), phospholipase (EC 3.1.1.4), ethanolaminephosphotransferase (EC 2.7.8.1), and cholinephosphotransferase (EC 2.7.8.2) are upregulated and downregulated upon phosphate depletion and restoration, respectively (see Figure 6). Whereas betaine lipid synthase BTA1 is upregulated and downregulated under phosphorus limitation and replenishing, respectively. These transcriptomic analyses are consistent with the level change of phospholipids obtained from lipidomic analysis (see Figure 3). 

We have noted that, Muhlroth et. al. [4] find that levels of not only the non-plastid phosphorus-free membrane lipid DGTS, but also the photosynthetic or plastid phosphorus-free lipids DGDG, MGDG, and SQDG are increased upon phosphate limitation in *N. oceanica* CCMP1779, ever though the photosynthetic efficiency is decreased by ~13% [4]. This result would suggest that level of the photosynthetic or plastid phosphorus-free membrane lipids is not necessarily correlated with that of photosynthetic efficiency.

*Nannochloropsis* sp. PJ12 produces EPA, whose level is reduced under phosphate limited condition (see Figure 2B). It also has been shown that the decreased EPA level is associated with the downregulation of enzymes involved in PUFA biosynthetic pathway upon nitrogen limitation [33]. However, under phosphate limitation, enzymes involved in the PUFA biosynthetic pathway are not concordantly downregulated, reminiscent to that of glycolysis and TCA cycle. These results would suggest that coordinated transcriptional alteration in a pathway appears to be disrupted upon phosphate limitation. This phenomenon could be attributed to the disruption of phosphorus-dependent signaling systems involved in transcriptional regulation of metabolic pathways.

In conclusion, we show that under phosphorus stress, levels of plastid membrane lipids DGDG, MDGD, PG, and SQDG and major non-plastid membrane lipids PC and PE are decreased, while level of non-plastid phosphorus-free betaine lipid DGTS is increased in *Nannochloropsis* sp. PJ12. Transcriptional level changes of respective enzymes are consistent with the level change of various lipid classes observed. Given the downregulation of enzymes involved in elongation cycle of FASII upon phosphorus stress, we propose that biosynthesis of betaine lipid DGTS and neutral lipid TAG via “remodeling” rather than “switching” mechanism. Hence, our results provide insight into mechanisms for cellular response to phosphorus stress in marine alga *Nannochloropsis* sp. PJ12.

## 4. Materials and Methods

### 4.1. Algal Strain and Culture Manipulation

The *Nannochloropsis* sp. PJ12 were previously isolated from the area of Pengjing city, Liaoning province, China [33]. It was cultivated modified f/2 medium (in 1 L, it contains 30 g sea salt, 150 mg NaNO_3_, 10 mg NaH_2_PO_4_·H_2_O, 3.15 mg FeCl_3_·6H_2_O, 4.16 mg Na_2_EDTA·2H_2_O, 10 μg CuSO_4_·5H_2_O, 6 μg Na_2_MoO_4_·2H_2_O, 22 μg ZnSO_4_·7H_2_O, 10 μg CoCl_2_·6H_2_O, and 180 μg MnCl_2_·4H_2_O, 2.5 μg Vitamin B_12_, 2.5 μg biotin, and 0.5 μg thiamine HCl) [32] in glass flask with orbital shaking at 100 rpm, 25 °C under continuous illumination of 50 μM photon m^−2^ s^−1^. Cell density was determined gravimetrically: 50–100 mL culture was first collected on filter paper, oven-dried overnight, and weighted on a balance (AG204, Mettler-Toledo Inc., Columbus, OH, USA). Alternatively, cell number was counted using the Neubauer chamber/hemocytometer (Hausser Scientific, Horsham, PA, USA). 

For phosphate-depletion treatment, 400 mL log-phase growth cells (~0.7 g/L) in f/2 medium (i.e., phosphate-replete) was harvested and half portion was resuspended in f/2-P (i.e., phosphate depleted) medium and the other half was resuspended in f/2-P medium as control. Cells at three days after growth in f/2-P medium (PD3) and in f/2 medium (PR3) were collected for analysis. For phosphate-restoration treatment, PD3 cells were shifted to f/2 medium and continued to growth for three days (PDR3). Sample PR3, PD3, and PDR3 were subjected to RNA-seq analysis and lipidomics analysis in triplicate.

### 4.2. Analysis of Total Phosphorus Content in Medium and Cell Biomass

To determine total phosphorus contents, the Total Phosphorus Test Kit (Lohand Biological, Hangzhou, China) was utilized. In brief, for determination of cell biomass phosphorus content, two aliquots of 50–100 mL of culture cells were harvested. One aliquot was dried for estimation of cell dry weight (CDW). The other aliquot was subjected to acid hydrolysis for orthophosphate conversion: Cell pellet was resuspended in 5% potassium persulfate to a final volume of 50 mL. Subsequently 4 mL of sulfuric acid was added and autoclaved at 120 °C for 30 min. The resulting solution was cooled and ready for determination of cell biomass-originated phosphorus. Total phosphate (TP) in sample solutions and standard solutions was determined using ammonium molybdate-ascorbic acid method in which the absorbance of molybdenum produced after reduction of phosphomolybdic acid by ascorbic acid was measured by the Lambda 465 spectrophotometer (PerkinElmer, Waltham, MA, USA) at a wavelength of 880 nm [42].

### 4.3. PAM Fluorescence Analysis

Maximal photosystem II quantum yield (Fv/Fm) of cells under PR, PD, PDR conditions was determined using chlorophyll fluorimeter based on pulse amplitude modulation (PAM) technique (Maxi Imaging-PAM system, Heinz Walz GmbH, Effeltrich, Germany) by following the manufacturer’s instruction. 

### 4.4. Transmission Electron Microscopy

Cells were harvested from approx. 15 mL culture prior to and after phosphate deprivation and phosphate restoration and washed with phosphate buffer or PB (in 100 mL, 530 mg of NaH_2_PO4·H_2_O, 165 mg of Na_2_HPO_4_·6H_2_O, pH7.0) three times. The washed cells were first fixed in 2.5% glutaraldehyde in PB for at least 4 h. After washing with PB, cells were fixed with 1% OsO_4_ in PB for 1–2 h and washed three times with PB for 15 min at each step. The fixed cells were dehydrated by a graded series of ethanol (30%, 50%, 70%, 80%, 90%, and 95%) for about 15 min at each step. Subsequently, cells were dehydrated by pure alcohol for 20 min. In the end, cells were transferred to absolute acetone for 20 min. Dehydrated cells were embedded in Spurr resin (Ted Pella, Inc., Redding, CA, USA) by mixing to a graded series of acetone and Spurr resin mixture (1:1 and 1:3) for 1 h at room temperature and then to the 100% Spurr resin mixture for overnight. Embedded cells were heated at 70 °C for 9 h prior to ultrathin sectioning using LEICA EM UC7 ultratome (Leica microsystems Ltd., Wetzlar, Germany). The resulting sections were stained by uranyl acetate and alkaline lead for 5 min and 10 min, respectively. Sections were analyzed using the Hitachi H-7650 instrument (Hitachi High-Technologies Co. Ibaraki, Japan).

### 4.5. Lipid Extraction and Fatty Acid Methyl Ester (FAME) Preparation

Two aliquots of 50–100 mL of algal culture were subjected to centrifugation at 3500× *g* for 5 min. Pellet of one aliquot was resuspended in 100 μL distill water, transferred to a filter paper, and dried in oven for determine the cell dry weight. Total lipid preparation followed the method by Bligh and Dyer with some modification [43]. In brief, cell pellet of the other aliquot was resuspended using 200 μL PBS and transferred to a mortar (Siqi abrasive materials Co. Lte. Jiaxing, China) for grinding. The ground cells were transferred to a centrifuge tube, triheptadecanoin was spiked as internal standard, 200 μL chloroform-methanol (1:2 vol:vol) solution was added and mixed thoroughly, and aqueous and organic phases were separated by centrifugation. The organic phase was transferred to a glass tube and dried with a flow of nitrogen gas. It resulted the total lipids. Lipid transesterification followed the method by van Wijngaarden [44]. In brief, total lipid was dissolved in 2 mL methanol containing 0.5 mol/L NaOH and transferred into a round-bottom flask for fraction distillation. The flask was heated in a water bath for 5 min, then added 2 mL methanol containing 14% BF_3_ and continue to heat at water bath. Approx. 2 mL hexane and NaCl saturated water was added in a flat-bottom flask containing distilled liquid, mixed well, and stand still until separation of organic and aqueous phases. The organic phase was transferred to a glass tube and FAME was ready after solvent evaporation was weighted using the AG204 balance (Mettler-Toledo Inc., Columbus, OH, USA). FAME content, FAME weight/CDW was determined in triplicate.

### 4.6. GC and GC-MS Analyses

GC analysis was applied to quantify various fatty acid molecular species as FAMEs once they were determined using GC-MS analysis. To determine the FAME species, 1 μL FAMEs was directly injected into the injection port of gas chromatograph (Shimadzu 2010Plus GC system, Shimadzu Co., Tokyo, Japan) coupled with a mass spectrometer system (MS) (Shimadzu QP2020 with quadrupole analyzer). The GC was operated on an Rtx-5MS GC column (30 m × 0.25 mm, id. with 0.25 μm film thickness of 5%-phenyl-methylpolysiloxane) (Restek Co., Bellefonte, PA, USA) and helium (purity 99.999%) was used as the carrier gas. The temperature of the injection port was set to 260 °C while the sample injection was made in splitless mode with a purge flow 50 mL min^−1^ for 1 min. The temperature program was started with an initial temperature at 160 °C, then 2 °C min^−1^ to 230 °C for 10 min. The mass spectrometer was operated in electron ionization (EI) mode with the ion source temperature at 230 °C. The electron energy was 70 eV. Full-scan MS data were acquired in the range of 50–500 *m*/*z* to obtain the fragmentation spectra of FAMEs. The LabSolutions (Shimadzu Co., Tokyo, Japan) was used to determine all the peaks in raw GC chromatogram. Library search was done for all the peaks using the National Institute of Standards and Technology NIST/EPA/NIH (NIST 14 Library).

To relatively quantify various FAME species, 1 μL of FAMEs containing methyl heptadecanoate derived from the spiked triheptadecanoin as internal standard was directly injected into the injection port of gas chromatograph (Shimadzu Co., Tokyo, Japan) equipped with flame ionization detector (FID) and Rtx-5 column (30 m × 0.32 mm, id. with 0.25 mm film thickness) (Restek Co., Bellefonte, PA, USA). The sample injection was made in split mode and the split ratio was 20:1. The temperature of the injection port and detector temperature were set to 260 °C and 230 °C, respectively. The temperature program was started with an initial temperature at 160 °C, then 2 °C min^−1^ to 230 °C for 10 min. Nitrogen was used as carrier gas, and its flow rate was 30 mL min^−1^. Hydrogen gas flow rate and air flow rate were 40 mL min^−1^ and 400 mL min^−1^, respectively.

### 4.7. LC-MS/MS-Based Lipidomic Analysis

The lipidomic analysis in this study adopted the method used by Nguyen et al. [45] and Legeret et al. [17] in which no internal standard was used and the relative quantitation of individual lipid species was achieved with Multiquant software (Applied Biosystems Sciex Pte Ltd., Redwood, CA, USA) based on the intensity values of extracting masses of different lipids identified previously. Relative quantity of lipid classes is estimated based on the sum level of all lipid species within the class. In brief, to determine lipid classes and the associated fatty acid species, 10 mg freeze-dried PJ12 cell sample were used to extract lipids as above mentioned. Lipidomic analysis was performed with a mass spectrometer equipped with a RS 3000 UPLC system (Thermo Orbitrap Elite, Thermo Fisher, Waltham, MA, USA). The spray capillary voltage was 3.2 KV for the negative ion mode and 3.0 KV for the positive ion mode. Lipid extracts were separated at 45 °C on a Kinetex C18 column (1.9 mm, 2.10 × 100 mm, phenomenex) for positive- and negative-mode mass spectrometry analysis. The mobile phases were acetonitrile: water (60:40) (A) and acetonitrile: isopropanol (10:90) (B) containing 0.1% formic acid and 10 mM ammonium acetate; the LC gradients were as follows: 0 min, 70% A and 30% B; 2 min, 70% A and 30% B; 20 min, 0A and 100% B; 40 min, 0% A and 100% B; 40.01 min, 70% A and 30% B; and 45 min, 70% A and 30% B. The flow rate was 0.4 mL min^−1^. Relative quantification was achieved with LipidSearch 4.2 (Thermo Fisher Scientific Inc., San Jose, CA, USA) on the basis of intensity values of extracting masses of different lipids previously identified.

TG and DG as [M + NH4]^+^ ions at a positive mode, while DGDG, DGTS, FA, LPE, LPG, MGDG, PA, PE, PG, PI, SQDG in the form of [M - H]^-^ ions and LPC and PC in the form of [M + HCOO]^−^ ions at a negative mode. Precursor ion and neutral loss scanning modes were employed to identify lipid species by the LipidSearch. For most lipid classes, fatty acid at the *sn*1 or *sn*2 position of the glycerol backbone was determined by following the published methods [30,46]. For some undetermined species, fatty acids at the *sn*-positions were estimated by comparing the relative intensities of the MS/MS fatty acid fragments to those of the characterized species of the same class under the same LC-MS/MS conditions. That is, the regiospecific preference of acyl groups in the glycerol backbones of lipids is determined based on the preferential loss of the acyl group in the *sn*1-position [47]. A list of 15 lipid classes and their levels consisting of 95 lipid species is available in Table S1.

### 4.8. Transcriptomic Analysis

Cell samples (PR3, PD3, and PDR3) in triplicate were subjected to RNA-seq analysis with a PE150 protocol (BGI, Shenzhen, China). A total of 18 Gigabase sequence data (2 Gigabases per sample) were obtained. The original datasets were deposited to the NCBI GEO database with an accession number of GSE124051 (https://www.ncbi.nlm.nih.gov/geo/query/acc.cgi?acc = GSE124051).

Raw data were filtered using FastQC [48] and low quality sequences was removed using Trimmomatic [49]. By using Trinity [50], high quality reads were assembled into a transcriptome containing 7140 ESTs or genes (see Table S2) after removing redundant genes by CD-Hit [51] and annotation using BLASTX against the “best” protein sequences from comprehensively annotated algal genomes of the green algae (Chlorophyta): *Chlamydomonas reinhardtii* [20], *Micromonas pusilla* [52], *Volvox carteri* [53], *Ostreococcus lucimarinus* [54], and *Coccomyxa subellipsoidea* [55]. Differentially transcribed genes were estimated using RSEM [56] and EdgeR [57] based on a cutoff of level change > 2-fold and FDR-corrected *p*-value < 0.05. Description of enzymes and the EC numbers were listed in Table S9.

### 4.9. Quantitative Real-Time PCR Assay

To validate the quality of RNA-seq-based transcription levels, quantitative real-time (qRT)-PCR assay was performed. For this reason, seven genes with different levels of TPM based on RNA-seq analysis were selected. The primers used were listed in Table S8. qRT-PCR was conducted as follows: Total RNA of 500 ng was used as template for reverse synthesis of the complimentary strand or cDNA with PrimeScript RT reagent kit plus gDNA Eraser (Takara Bio Inc., Tokyo, Japan) according to manufacturer’s instruction. The resulting cDNA was tenfold diluted and subjected to qRT-PCR analysis using SYBR Premix Ex Taq II kit (Takara Bio Inc., Shiga, Japan) on a ABI 7300 instrument (Thermo Fisher Scientific, Waltham, MA, USA). The PCR condition was as follows: Initial denaturing for 2 min at 95 °C, followed by 35 cycles of 15 s at 95 °C and 1 min at 60 °C. Housekeeping genes (exportin 1, nuclear transport, actin) were based on the study by Muhlroth et al. [4]. Correlations between RT-PCR and RNA-seq were analyzed. Relative changes of gene transcription levels after PD or PDR tested using qRT-PCR and RNA-seq methodologies are highly correlated with a correlation coefficient of 0.946 (see Table S8).

### 4.10. Statistical Analysis

A hypergeometric test [58] was used to determine the statistical significance of enrichment for GO functions and KEGG pathways in a subset of genes when compared to the whole genome/transcriptome. The Bonferroni correction [59,60] was applied in all multiple tests in this study. Differences between means of two and more subgroups are tested using t-test and one-way analysis of variance (ANOVA), respectively.

## Figures and Tables

**Table 1:** Enriched functions in group of genes inversely and positively correlated with phosphate concentrations.

(A) Inversely correlated genes				
**Pathway**	**G1-5 ^a^**	**All ^b^**	**FE ^c^**	***p*-Value**
Cysteine and methionine metabolism	6	51	2.46	0.038
(B) Positively correlated genes				
**Pathway**	**G6-10 ^d^**	**All ^b^**	**FE ^c^**	***p*-Value**
Val, leu and ile degradation	49	49	28.91	2.20 × 10^16^
Amino sugar metabolism	41	41	28.91	2.20 × 10^16^
Glutathione metabolism	33	33	28.91	2.20 × 10^16^
Citrate cycle	32	32	28.91	2.20 × 10^16^
Propanoate metabolism	28	28	28.91	2.20 × 10^16^
Sulfur metabolism	23	23	28.91	2.20 × 10^16^
Steroid biosynthesis	23	23	28.91	2.20 × 10^16^
Starch and sucrose metabolism	20	20	28.91	2.20 × 10^16^
Pantothenate and CoA biosynthesis	20	20	28.91	2.20 × 10^16^
Selenocompound metabolism	20	20	28.91	2.20 × 10^16^

^a^, pathway-associated gene numbers in a subset of 342 inversely correlated genes; ^b^, pathway-associated gene numbers in a total of 7140 genes; ^c^, FE for fold enrichment (*p*-value < 0.05); ^d^, pathway-associated gene numbers in a subset of 247 positively correlated genes.

## Supplementary Materials

The following files are available online at https://www.mdpi.com/1660-3397/17/9/519/s1: Table S1: Lipidomic profiles of *Nannochloropsis* sp. PJ12 under PR, PD, and PDR conditions. Table S2: Transcriptomic profiles of *Nannochloropsis* sp. PJ12 under PR, PD, and PDR conditions. Table S3: K-mean 10-group analysis of common DE genes upon phosphorus depletion and replenishment. Table S4: Numbers of genes associated with KEGG pathways. Table S5: Transcriptional profiles of enzymes involved in Calvin cycle, glycolysis, and TCA cycle based on the sum level of multiple-copy genes when exist. Table S6: Transcriptional profiles of enzymes involved in FASII, GLSp, GLSc, and beta-oxidation based on the sum level of multiple-copy genes when exist. Table S7: Transcriptional profiles of enzymes involved in PUFA synthesis based on the sum level of multiple-copy genes when exist. Table S8: Correlation between qRT-PCR and RNA-seq analyses. Table S9: List of enzymes and EC numbers used in the study.
